# Systemic inflammatory indices mediate the association between hyperuricemia and left ventricular hypertrophy: evidence from a single-center retrospective cross-sectional study

**DOI:** 10.3389/fendo.2025.1742938

**Published:** 2026-01-05

**Authors:** Jingyuan Li, Yang Xu, Ruting Li, Xin Huang, Shuhui Hu, Fei Yan, Ying Gong, Xiaoqing Zhang, Fengyao Sun, Lei Chen, Ying Chen

**Affiliations:** 1 Department of Endocrinology and Metabolism, The Affiliated Hospital of Qingdao University, Qingdao, China; 2 Department of Cardiovascular Medicine, The Affiliated Hospital of Qingdao University, Qingdao, China; 3 School of Health and Life Sciences, University of Health and Rehabilitation Sciences, Qingdao, China

**Keywords:** hyperuricemia, left ventricular hypertrophy (LVH), systemic immune-inflammation index (SII), systemic inflammation, systemic inflammation response index (SIRI)

## Abstract

**Aims:**

Hyperuricemia (HUA) is associated with left ventricular hypertrophy (LVH), a reversible marker of cardiac injury. Systemic inflammation drives ventricular remodeling, and the systemic immune-inflammation index (SII) and systemic inflammation response index (SIRI) may reflect this process. This study investigated their associations with LVH in patients with HUA.

**Methods:**

We analyzed 3, 632 patients with HUA hospitalized between 2014 and 2024, excluding those with prior hypertension, diabetes, or advanced chronic kidney disease. Baseline demographic, biochemical, and echocardiographic data were collected. LVH was defined by sex-specific left ventricular mass index thresholds. SII and SIRI were calculated, log-transformed, and analyzed by k-means clustering. Associations with LVH were assessed using logistic regression, restricted cubic spline (RCS), and threshold effect models. Mediation analysis evaluated their role between serum urate and LVH.

**Results:**

Among all patients, 452 (12.5%) had LVH. Compared with non-LVH patients, those with LVH were older, more often female, and exhibited higher systolic blood pressure, serum urate, glucose, SII, and SIRI, and lower eGFR and LVEF (all p < 0.05). Elevated SII, SIRI, and high inflammatory patterns were independently associated with LVH (all p < 0.01). RCS revealed a nonlinear “J-shaped” association for lnSII with a threshold at 5.99, while lnSIRI showed a linear dose–response. Mediation analysis indicated systemic inflammation partially mediated the urate–LVH relationship (11.9% for lnSII, 29.9% for lnSIRI).

**Conclusion:**

In HUA patients, SII and SIRI are positively correlated with LVH and partially mediate the relationship between urate and cardiac remodeling, emphasizing the role of systemic inflammation in hyperuricemia related cardiovascular diseases.

## Graphical Abstract

**Figure:**
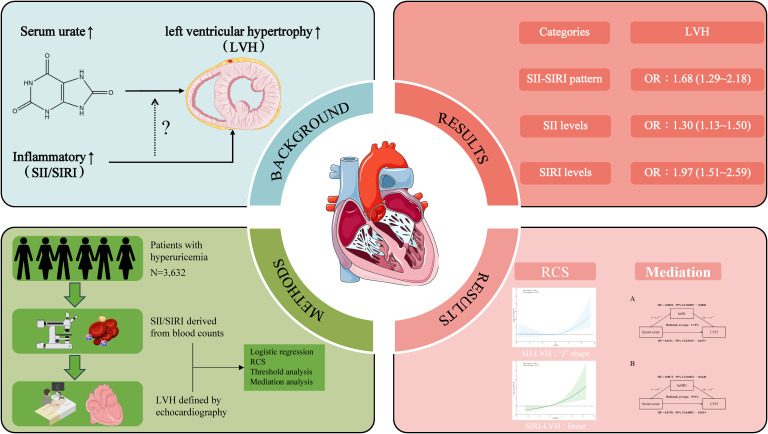
Conceptual overview of the association between systemic inflammation and left ventricular hypertrophy (LVH) in patients with hyperuricemia (HUA).

## 1 Introduction

Hyperuricemia (HUA) serves as the biochemical foundation of gout and is strongly linked to structural and functional abnormalities of the cardiovascular system (1–3). Increasing evidence suggests that patients with HUA are more likely to develop left ventricular hypertrophy (LVH), diastolic dysfunction, and heart failure (HF) (4–6). As a key indicator of target organ injury, LVH is not only an independent risk factor for cardiovascular events and all-cause mortality (7) but the only reversible indicator whose regression can significantly improve cardiovascular outcomes as well (8). Evidence has shown that in patients with LVH, a reduction in left ventricular mass (LVM) is associated with a series of favorable pathophysiological changes, including improved systolic function and diastolic filling, increased coronary flow reserve, reduced ventricular arrhythmias, and a potential prevention of atrial fibrillation (9). Therefore, the early clinical identification of LVH holds significant clinical value in the prevention of maladaptive ventricular remodeling, HF, and subsequent cardiovascular complications in patients with HUA.

Although HUA has been shown to induce LVH (6, 10, 11), clinical evidence indicates that the cardiovascular benefits of various xanthine oxidase inhibitors (XOIs) are inconsistent (11), implying that alterations in serum uric acid levels alone may be insufficient to account for ventricular remodeling in HUA patients. Consequently, identifying determinants of LVH within the HUA patients has become an emerging focus of research. Comprehensive insight into these risk factors and their interplay with LVH is crucial for clarifying the underlying pathophysiological mechanisms and for formulating precise prevention and intervention strategies.

Previous studies have confirmed the predictive value of inflammatory cytokines (such as TNF-α, IL-6, and IL-1β) in cardiovascular diseases (CVD) and HF (12–16). Furthermore, evidence suggests that serum urate enhances the release of proinflammatory cytokines through upregulation of urate transporter 1 (URAT1) and glucose transporter 9 (GLUT9), consequently activating the NF-κB/MAPK signaling cascade and leading to myocardial hypertrophy and fibrosis (17). Accordingly, we postulate that inflammatory cytokines may also be involved in the pathogenesis of LVH in patients with HUA. However, the high cost of traditional inflammatory cytokines assays limits their widespread use in clinical practice (18). In contrast, the systemic immune-inflammation index (SII) and systemic inflammation response index (SIRI), calculated from peripheral blood cell counts, can reflect the immune status and systemic inflammatory burden of the body, and have shown promising clinical and experimental utility (19). Evidence indicates that SII and SIRI, as markers of inflammation, are strongly correlated with the occurrence and clinical outcomes of multiple disorders, such as heart failure, stroke, and psoriasis (20–24). However, the association between SII/SIRI and LVH in patients with HUA remains unclear, and the dose–response relationship or mediating effects of these inflammatory markers in the development of LVH among HUA patients are still lacking.

Based on the above background, this study aimed to investigate the relationship between inflammatory markers SII/SIRI and LVH in patients with HUA, providing new insights into the link between HUA and cardiovascular diseases.

## 2 Materials and methods

### 2.1 Study subjects and methods

We conducted a single-center retrospective cross-sectional study including 3, 632 patients with HUA who were hospitalized at the Affiliated Hospital of Qingdao University between January 1, 2014 and December 31, 2024 (**Supplementary Figure 1**). Inclusion criteria were as follow: (1) a prior diagnosis of HUA with serum uric acid levels >8 mg/dL upon admission; and (2) completion of transthoracic echocardiography upon admission. Exclusion criteria were as follow: (1) echocardiographic reports lacking quantitative values; (2) age <18 or >80 years; (3) prior diagnosis of gout; (4) pre-existing diagnosis of diabetes or hypertension, or chronic use of related medications; (5) presence of malignancy, hematological disorders, congenital heart disease, or autoimmune diseases; (6) history of coronary artery disease or other structural heart diseases; (7) missing baseline information; and (8) chronic kidney disease stage >3. Baseline data collected at admission included age, smoking and alcohol consumption history, height, weight, systolic and diastolic blood pressure, and body mass index (BMI). Blood samples were collected within 24 hours of admission to assess hematologic and biochemical parameters, including white blood cell (WBC), neutrophil (NBC), lymphocyte (LBC), Monocyte, and peripheral platelet (PLT), alanine aminotransferase (ALT), aspartate aminotransferase (AST), triglycerides (TG), total cholesterol (TC), fasting blood glucose (FBG), serum creatinine, and serum urate. Transthoracic echocardiography was performed using a PHILIPS EPIQ 5 system by experienced sonographers according to current recommendations of the American Society of Echocardiography and the European Association of Cardiovascular Imaging. Aortic diameter (AO), left atrial diameter (LA), interventricular septum (IVS), left ventricular posterior wall thickness (LVPWT), left ventricular end-diastolic diameter (LVEDD) and left ventricular ejection fraction (LVEF) were measured in the parasternal long-axis view using M-mode or two-dimensional guided techniques. BMI = weight (kg)/height² (m²), SII = platelet count × neutrophil count/lymphocyte count. SIRI = monocyte count × neutrophil count/lymphocyte count. Left ventricular mass (LVM) was calculated as: 0.8 × [1.04 × (IVS + LVPWT + LVEDD)³ − LVEDD³] + 0.6. Left ventricular mass index (LVMI) was obtained by dividing LVM by body surface area (BSA). BSA was estimated using the formula: BSA = 0.0061 × height (cm) + 0.0128 × weight (kg) − 0.1529.

The study complied with the Declaration of Helsinki, which was approved by the Ethics Committee of the Affiliated Hospital of QingDao University (QYFYWZLL26144).

### 2.2 Classification criteria

HUA was defined as measurements of fasting serum urate levels ≥7 mg/dL in adults. Left ventricular hypertrophy was defined as a left ventricular mass index (LVMI) >125 g/m² in males and >120 g/m² in females. Hypertension on admission was defined as systolic blood pressure (SBP) ≥140 mmHg and/or diastolic blood pressure (DBP) ≥90 mmHg.

### 2.3 Definitions and diagnostic criteria

Participants were stratified into LVH and non-LVH groups according to whether left ventricular hypertrophy was present. Subgroup analyses were conducted by age (18–65 vs. ≥65 years), gender (male/female), smoking status (Never smoking/Ever smoking), drinking status (Never drinking/Ever drinking), BMI categories (≤24, 24–28, ≥28 kg/m²), hypertension status (No/Yes), CKD stage (I/II vs. III), FBG levels (<6.1 vs. ≥6.1mmol/L), and serum urate levels (<8 vs. ≥8 mg/dL).

### 2.4 Data analysis

Statistical analyses were performed using R software version 4.4.3. The distribution of continuous variables was evaluated using histograms and Q–Q plots (**Supplementary Figures 3**, **4**). Given the large sample size, minor deviations from perfect normality were expected and formal tests of normality (e.g., Shapiro–Wilk) were not used as the sole criterion for selecting statistical tests, because they tend to be overly sensitive in large samples. Variables that were approximately symmetric without marked skewness were summarized as mean ± standard deviation (SD) and compared using independent-sample t-tests, whereas clearly skewed variables were presented as median (interquartile range, IQR) and compared using the Mann–Whitney U test. Categorical variables were presented as counts and percentages, and comparisons between groups were conducted using the chi-square test.

Clustering analysis was performed based on SII and SIRI. The SII and SIRI levels were normalized prior to analysis using the k-means clustering algorithm. K-means clustering is a non-parametric algorithm that assigns samples to clusters by minimizing the squared Euclidean distance between each sample vector and the centroid of its designated cluster. The elbow method was employed to identify the optimal number of clusters. Dimensionality reduction and visualization of subgroups were conducted using t-distributed stochastic neighbor embedding (t-SNE) (25, 26) (**Supplementary Figure 2**).

Furthermore, SII and SIRI levels were divided into tertiles, classifying individuals into low, middle, and high groups for stratified analysis. Univariable and multivariable logistic regression models were used to evaluate the associations between SII-SIRI patterns (as defined by k-means clustering), SII or SIRI levels, and the risk of LVH. Given the right-skewed distribution of SII, SIRI and serum urate, log-transformed variables were used in regression models to examine their relationship with LVH risk as continuous predictors.

The crude model included no covariate adjustment, while the fully adjusted model controlled for potential confounders including age, gender, BMI, drinking history, smoking history, SBP, DBP, serum creatinine, FBG, and serum urate. Covariates were selected *a priori* based on their clinical relevance and previously reported associations with both systemic inflammation and cardiac remodeling, as well as their potential role as confounders in the relationship between SII/SIRI and LVH. To assess multicollinearity among the independent variables, we calculated variance inflation factors (VIFs) for all covariates included in the multivariable models. Restricted cubic spline (RCS) analysis was applied to flexibly explore the potential non-linear association between inflammatory indices (lnSII or lnSIRI) and the risk of LVH. Knots were placed at the 10th, 50th, and 90th percentiles of the distribution. Both overall and non-linear P values were calculated to evaluate the shape of the relationship. Threshold effect analysis was then performed using two-piecewise linear regression models to further assess potential inflection points suggested by the RCS curves. The optimal cut-off values were determined by likelihood ratio tests comparing the one-line linear regression model with the two-piecewise regression model. To investigate the potential mechanisms of SII and SIRI in left ventricular remodeling among patients with HUA, mediation analysis was performed to assess their potential mediating effects between elevated serum uric acid and left ventricular remodeling. In these models, we adjusted for the same set of covariates as in the multivariable models, except that serum urate was not included as an adjustment variable to avoid over-controlling the exposure.

Patients with missing values for key baseline variables were excluded from the analysis; thus, all analyses were based on complete cases without imputation.

## 3 Results

### 3.1 Participants characteristics

A total of 3, 632 patients with HUA were enrolled, including 452 (12.45%) with LVH (**Table 1**). Compared with non-LVH patients, those with LVH were older, more often female, and had higher rates of smoking and drinking (all p < 0.001). They also exhibited higher SBP, SII, SIRI, ALT, serum urate, FBG, and neutrophil counts, but lower eGFR and lymphocyte counts (all p < 0.05). No significant differences were observed in BMI, DBP, serum creatinine, triglycerides, or platelet counts. Compared with the non-LVH group, the LVH group exhibited significantly greater LA, LVEDD, IVS and LVPWT. In contrast, LVEF was significantly lower in the LVH group (P < 0.001).

**Table 1:** Baseline characteristics of participants (n = 3, 632).

Variables	Non-LVH (n = 3180)	LVH (n = 452)	*P*
Age, years, M (Q_1_, Q_3_)	64.00 (53.00, 71.00)	70.00 (60.75, 75.00)	**<0.001**
Gender, n(%)			**<0.001**
Female	603 (18.96)	243 (53.76)	
Male	2577 (81.04)	209 (46.24)	
Marriage, n(%)			**<0.001**
Never marriage	203 (6.38)	8 (1.77)	
Ever marriage	2977 (93.62)	444 (98.23)	
Smoking, n(%)			**<0.001**
Never smoking	1363 (42.86)	98 (21.68)	
Ever smoking	1817 (57.14)	354 (78.32)	
Drinking, n(%)			**<0.001**
Never drinking	1253 (39.40)	101 (22.35)	
Ever drinking	1927 (60.60)	351 (77.65)	
BMI, kg/m^2^, Mean ± SD	25.86 ± 4.18	25.87 ± 4.42	0.946
SBP, mmHg, Mean ± SD	131.29 ± 17.04	139.95 ± 21.50	**<0.001**
DBP, mmHg, Mean ± SD	80.41 ± 11.51	81.18 ± 13.94	0.266
SII, 10^9^ cells/L, M (Q_1_, Q_3_)	397.19 (275.88, 610.86)	452.96 (295.14, 704.48)	**<0.001**
SIRI, 10^9^ cells/L, M (Q_1_, Q_3_)	0.87 (0.58, 1.43)	1.02 (0.62, 1.88)	**<0.001**
ALT, U/L, M (Q_1_, Q_3_)	21.00 (14.90, 32.23)	18.00 (13.35, 29.00)	**<0.001**
AST, U/L, M (Q_1_, Q_3_)	19.00 (15.47, 25.00)	19.20 (15.82, 27.00)	0.306
Serum creatinine, μmol/L, M (Q_1_, Q_3_)	89.00 (74.00, 100.00)	86.00 (71.00, 102.00)	0.296
eGFR, ml/min/1.73/m^2^, M (Q_1_, Q_3_)	81.88 (68.78, 95.82)	80.75 (65.70, 92.67)	**0.010**
Serum Urate, mg/dL, M (Q_1_, Q_3_)	7.66 (7.30, 8.35)	7.83 (7.30, 8.57)	**0.003**
FBG, mmol/L, Mean ± SD	5.15 ± 0.94	5.28 ± 0.98	**0.005**
Triglyceride, mmol/L, M (Q_1_, Q_3_)	1.45 (1.00, 2.25)	1.43 (0.99, 2.16)	0.733
WBC, 10^9^ cells/L, M (Q_1_, Q_3_)	6.50 (5.34, 7.91)	6.53 (5.26, 8.48)	0.291
Neutrophil, 10^9^ cells/L, M (Q_1_, Q_3_)	3.66 (2.85, 4.87)	3.86 (2.89, 5.44)	**0.007**
Lymphocyte, 10^9^ cells/L, M (Q_1_, Q_3_)	1.95 (1.50, 2.46)	1.81 (1.28, 2.31)	**<0.001**
Monocyte, 10^9^ cells/L, M (Q_1_, Q_3_)	0.48 (0.37, 0.61)	0.49 (0.37, 0.64)	0.240
Platelet, 10^9^ cells/L, M (Q_1_, Q_3_)	218.00 (177.00, 260.00)	213.50 (163.00, 256.00)	0.054
AO, cm, Mean ± SD	2.39 ± 0.40	2.38 ± 0.18	0.440
LA, cm, Mean ± SD	3.57 ± 0.44	3.94 ± 0.55	**<0.001**
LVEDD, cm, Mean ± SD	4.55 ± 0.35	4.94 ± 0.50	**<0.001**
IVS, cm, Mean ± SD	1.02 ± 0.11	1.20 ± 0.23	**<0.001**
LVPWT, cm, Mean ± SD	0.94 ± 0.09	1.08 ± 0.15	**<0.001**
LVEF, %, Mean ± SD	0.63 ± 0.03	0.60 ± 0.07	**<0.001**

LVH, left ventricular hypertrophy; BMI, body mass index; SBP, systolic blood pressure; DBP, diastolic blood pressure; WBC, white blood cell; SII, systemic immune-inflammation index; SIRI, systemic inflammation response index; ALT, alanine aminotransferase; AST, aspartate aminotransferase; FBG, fasting blood glucose; eGFR, estimated glomerular filtration; AO, Aortic diameter; LA, left atrial diameter; LVEDD, left ventricular end-diastolic diameter; IVS, interventricular septum; LVPWT, left ventricular posterior wall thickness; LVEF, left ventricular ejection fraction. Bold values indicate statistical significance at p < 0.05.

Echocardiographic comparisons revealed that patients with a high SII–SIRI pattern exhibited significantly increased IVS, LVPWT, and LVMI, whereas their LVEF values were lower (all P < 0.05). Consistently, increased IVS, LVPWT, and LVMI, as well as reduced EF are observed in high SII/SIRI levels. However, no significant differences were observed among groups in AO, LA, or LVEDD (**Supplementary Table 1**).

### 3.2 Association between SII-SIRI pattern/SII/SIRI levels and LVH

The SII–SIRI pattern, SII level, and SIRI level were all positively associated with the risk of LVH (**Table 2**). In the crude model (Model 1), patients in the higher SII–SIRI cluster had a significantly increased risk of LVH compared to those in the lower cluster (OR = 1.68, 95% CI: 1.34–2.11, p < 0.001). After adjustment for demographic, lifestyle, and clinical covariates (Model 2), the association remained robust (OR = 1.72, 95% CI: 1.34–2.21, p < 0.001), and persisted after additional adjustment for serum creatinine, serum uric acid, FBG, and TG (Model 3: OR = 1.68, 95% CI: 1.29–2.18, p < 0.001).

**Table 2:** Odds ratios (95%CI) of LVH and SII-SIRI pattern/SII/SIRI levels in the patients (n = 3, 632) (10^9^ cells/L).

Categories	Model 1		Model 2		Model 3	
	OR (95%CI)	*P*	OR (95%CI)	*P*	OR (95%CI)	*P*
SII-SIRI pattern						
Low	1.00 (Reference)	Ref.	1.00 (Reference)	Ref.	1.00 (Reference)	Ref.
High	1.68 (1.34~2.11)	**<0.001**	1.72 (1.34~2.21)	**<0.001**	1.68 (1.29~2.18)	**<0.001**
SII levels						
Low	1.00 (Reference)	Ref.	1.00 (Reference)	Ref.	1.00 (Reference)	Ref.
Medium	1.38 (1.08~ 1.77)	**0.010**	1.36 (1.04~ 1.78)	**0.023**	1.44 (1.09~ 1.89)	**0.010**
High	1.52 (1.20~ 1.94)	**<0.001**	1.52 (1.17~1.97)	**0.002**	1.51 (1.15 ~ 1.99)	**0.003**
Continuous (lnSII)	1.29 (1.13~1.46)	**<0.001**	1.29 (1.13~1.49)	**<0.001**	1.30 (1.13~1.50)	**<0.001**
SIRI levels						
Low	1.00 (Reference)	Ref.	1.00 (Reference)	Ref.	1.00 (Reference)	Ref.
Medium	1.14 (0.88~1.48)	0.336	1.23 (0.93 ~1.63)	0.146	1.17 (0.87~1.57)	0.289
High	1.85 (1.47~2.34)	**<0.001**	2.00 (1.55~2.58)	**<0.001**	1.97 (1.51~2.59)	**<0.001**
Continuous (lnSIRI)	1.31 (1.17~1.46)	**<0.001**	1.41 (1.26~1.59)	**<0.001**	1.39 (1.22~1.58)	**<0.001**

Model 1: Crude.

Model 2: Adjust: AGE, Gender, Smoking, Drinking, BMI, SBP, DBP.

Model 3: Adjust: AGE, Gender, Smoking, Drinking, BMI, SBP, DBP, serum creatinine, serum urate, FBG, TG.

OR, Odds Ratio; CI, Confidence Interval; LVH, left ventricular hypertrophy; BMI, body mass index; SBP, systolic blood pressure; DBP, diastolic blood pressure; SII, systemic immune-inflammation index; SIRI, systemic inflammation response index; FBG, fasting blood glucose; TG, triglycerides.

Bold values indicate statistical significance at p < 0.05.

Similarly, compared with the low SII group, patients in the highest SII group had a significantly elevated risk of LVH (Model 3: OR = 1.51, 95% CI: 1.15–1.99, p = 0.003), and lnSII as a continuous variable showed a stable positive association (OR = 1.30, 95% CI: 1.13–1.50, p < 0.001). For SIRI, patients in the highest SIRI group also exhibited markedly increased LVH risk (Model 3: OR = 1.97, 95% CI: 1.51–2.59, p < 0.001), and lnSIRI was consistently associated with LVH as a continuous variable (OR = 1.39, 95% CI: 1.22–1.58, p < 0.001). No evidence of problematic multicollinearity was observed (all VIFs < 2.0) (**Supplementary Table 2**).

### 3.3 Dose-response relationship between SII/SIRI levels and LVH

**Figure 1:**
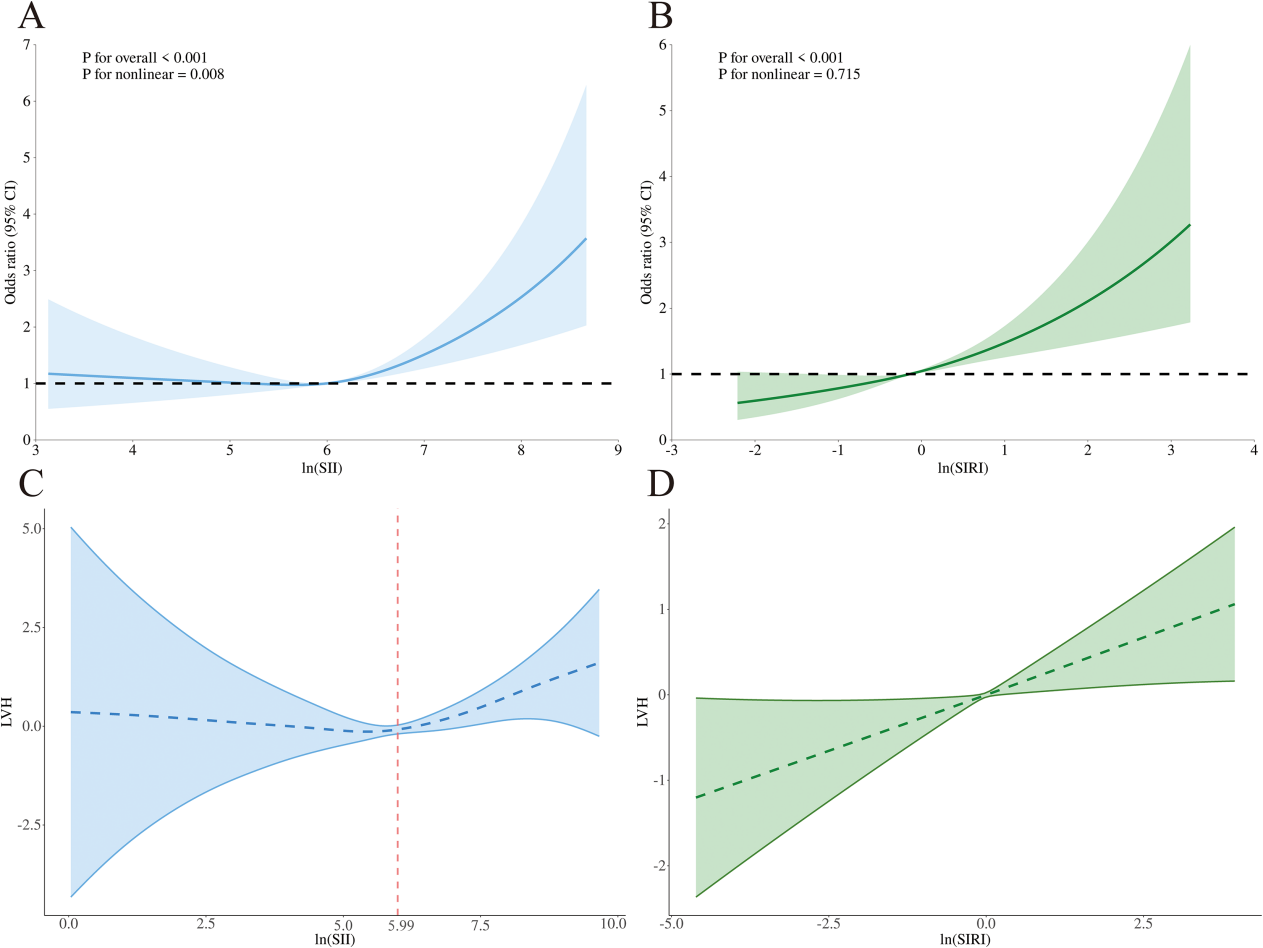
Restricted cubic spline (RCS) and threshold effect analyses for the associations of lnSII and lnSIRI with LVH. **(A)** RCS curve showing a non-linear “J-shaped” association between lnSII and LVH risk (overall p < 0.001, non-linear p = 0.008). **(B)** RCS curve indicating a linear association between lnSIRI and LVH risk (overall p < 0.001, non-linear p = 0.715). **(C)** Threshold effect analysis for lnSII with an inflection point at 5.99, above which the risk of LVH increased significantly. **(D)** Threshold effect analysis for lnSIRI with a suggested cut-off at –0.87, though no significant non-linear association was observed. SII, systemic immune-inflammation index; SIRI, systemic inflammation response index.

RCS analysis revealed a non-linear association between lnSII and LVH risk (overall p < 0.001, non-linear p = 0.008), with an inflection point around 5.99, and the curve is in a “J” shape (**Figure 1**). Below this threshold, lnSII was not significantly associated with LVH (OR = 0.97, 95% CI: 0.70–1.35, p = 0.868), whereas levels above 5.99 were linked to a substantially increased risk (OR = 1.66, 95% CI: 1.30–2.11, p < 0.001). The two-piecewise linear regression provided a significantly better fit than the linear model (LRT p = 0.034).

For lnSIRI, the relationship with LVH was linear overall (overall p < 0.001, non-linear p = 0.715) (**Figure 1**). A potential threshold at –0.87 was suggested, but the association below this value was not significant (OR = 2.46, 95% CI: 0.86–6.98, p = 0.092), while the risk remained significant above this level (OR = 1.37, 95% CI: 1.18–1.59, p < 0.001). However, the two-piecewise model did not improve the fit compared with the linear model (LRT p = 0.806).

### 3.4 Subgroup analysis

**Figure 2:**
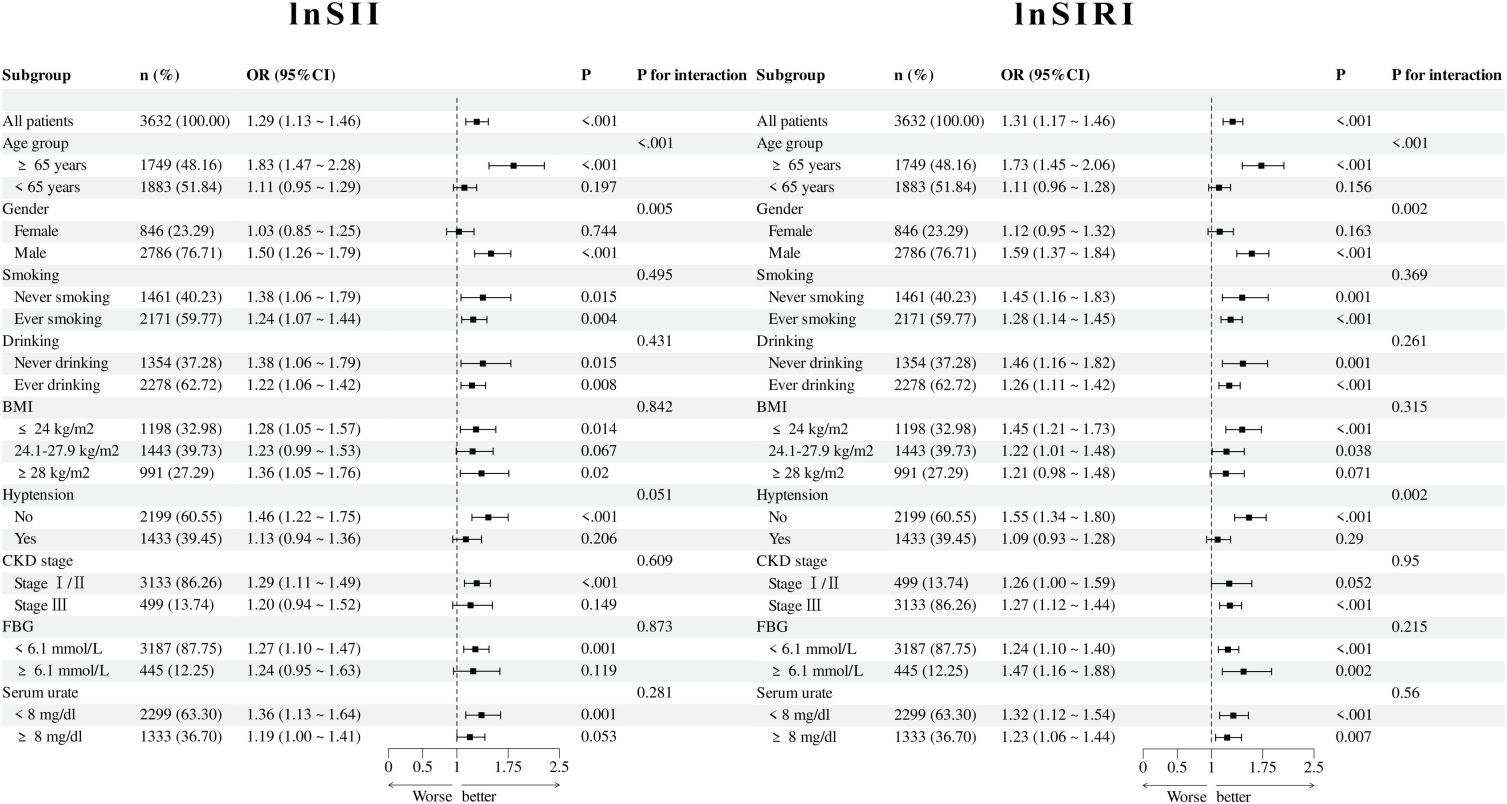
Subgroup analyses of the associations between lnSII and lnSIRI with left ventricular hypertrophy (LVH). Odds ratios (ORs) and 95% confidence intervals (CIs) were estimated using multivariable logistic regression across subgroups defined by age, sex, smoking, drinking, body mass index (BMI), hypertension, chronic kidney disease (CKD) stage, fasting blood glucose (FBG), and serum urate levels. Elevated lnSII and lnSIRI were significantly associated with LVH risk in most subgroups (all p < 0.05). Stronger associations were observed among older patients, males, those with lower BMI, and those without hypertension. Significant interactions were detected for age, sex, and hypertension, while no significant interactions were found for other stratifications.

To further explore the impact of potential confounding factors on the association between lnSII or lnSIRI levels and left ventricular hypertrophy (LVH), subgroup analyses were performed according to age, gender, smoking history, drinking history, BMI, hypertension, CKD stage, FBG, and serum urate levels (**Figure 2**). The results showed that elevated lnSII levels were significantly associated with an increased risk of LVH in several subgroups, with stronger associations observed in older, male, lower BMI, and those without hypertension (all p < 0.05). Similarly, lnSIRI levels were consistently associated with LVH risk across most subgroups (all p < 0.05). Significant interactions were observed for age, gender, and hypertension in lnSII or lnSIRI analyses, whereas no significant interactions were detected for other stratifications (all p for interaction > 0.05).

### 3.5 Mediation analysis

**Figure 3:**
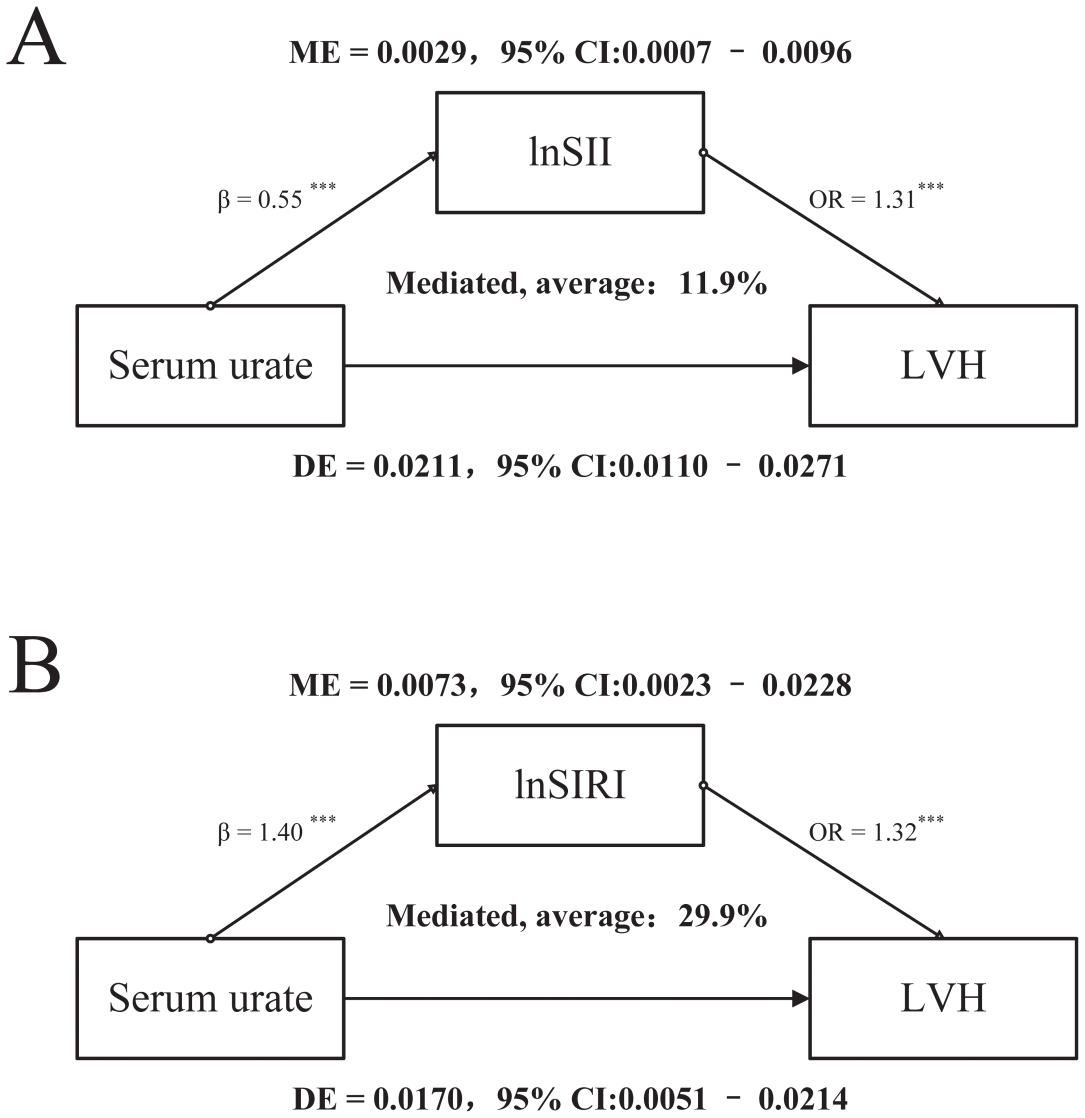
Mediation analysis of the association between serum urate, systemic inflammation, and left ventricular hypertrophy (LVH). **(A)** Mediation model with lnSII as the mediator. Serum urate was positively associated with lnSII (β = 0.55, ***p < 0.001), and lnSII was independently associated with LVH risk (OR = 1.31, ***p < 0.001). The indirect effect of serum urate on LVH through lnSII was significant, accounting for 11.9% of the total effect (ME = 0.0029, 95% CI: 0.0007–0.0096). **(B)** Mediation model with lnSIRI as the mediator. Serum urate was strongly associated with lnSIRI (β = 1.40, ***p < 0.001), and lnSIRI showed a stable association with LVH risk (OR = 1.32, ***p < 0.001). The mediated effect explained 29.9% of the total effect (ME = 0.0073, 95% CI: 0.0023–0.0228). ME, mediation effect; DE, direct effect; ***p < 0.001.

Mediation analyses were conducted using SII and SIRI as mediators separately (**Figure 3**). In both models, serum urate was significantly associated with LVH, with consistent evidence of a direct effect and a partial mediation through systemic inflammation. When lnSII was specified as the mediator, the proportion mediated was 11.9% (95% CI: 3.7%–33.1%, p < 0.001), whereas lnSIRI accounted for a larger mediated proportion of 29.9% (95% CI: 14.8%–79.5%, p = 0.0012). In addition, serum urate was positively associated with both inflammatory indices (lnSII: β = 0.55, 95% CI: 0.34–0.76; lnSIRI: β = 1.4, 95% CI: 1.17–1.62; all p < 0.001), and each index was independently related to LVH risk (lnSII: OR = 1.31, 95% CI: 1.14–1.50; lnSIRI: OR = 1.32, 95% CI: 1.17–1.48; all p < 0.001).

## 4 Discussion

In this single-center retrospective cross-sectional study conducted in a hospital-based population, we observed that about 12.5% of patients with HUA exhibited LVH, a prevalence that remained considerable even after excluding individuals with hypertension, diabetes, or advanced renal dysfunction (**Table 1**). This finding suggests that LVH may also represent an important clinical complication of HUA (6). Given the LVH, as a marker of cardiac target organ injury, is closely associated with adverse ventricular remodeling as well as an increased risk of heart failure, arrhythmias, and cardiovascular mortality (6, 15, 27), identifying markers for early risk stratification in the HUA population holds important clinical significance.

LVH represents a maladaptive remodeling process of the ventricle that occurs in response to both acute and chronic myocardial injury (27). In addition to traditional risk factors such as hypertension, diabetes, and obesity (8, 28), HUA has also been identified as an independent risk factor for LVH (1, 2, 6, 17). The potential mechanisms by which HUA induces LVH may include: (1) stimulation of the renin–angiotensin–aldosterone system (RAAS), inhibition of endothelial nitric oxide synthesis, and promotion of vascular smooth muscle cell proliferation, leading to microvascular injury (29). (2) activation of the NLRP3 inflammasome, resulting in elevated levels of inflammatory cytokines (such as TNF-α, IL-6, IL-1β, IL-18, and IL-8), which further induce cardiomyocyte apoptosis and necrosis, promote the development and progression of LVH and adverse ventricular remodeling, and increase the risk of HF (14, 27, 30–32). Given the activation of systemic inflammation, serum uric acid alone cannot fully predict the occurrence or prognosis of LVH. This may partially explain the inconsistent cardiovascular benefits observed in previous studies focusing solely on urate-lowering therapy (11). Consistently, accumulating clinical evidence indicates that IL-1β inhibition, through blockade of IL-1β–driven inflammatory cascades, markedly enhances cardiovascular prognosis (18, 33). Hence, it is important to monitor inflammation biomarkers and claim their involvement in myocardial remodeling in patients with HUA, with the aim of identifying potential targets for early intervention.

Despite their important role in the diagnosis and prognosis of ventricular remodeling, classical inflammatory markers are difficult to implement broadly in clinical practice owing to prolonged testing procedures and high expenses (18). Recently, new inflammation markers derived from peripheral blood cell counts, including SII, SIRI, NLR and etc., have been shown to capture both innate and adaptive immune responses (20, 22, 24). Numerous studies have demonstrated that markers such as SII and SIRI are strongly linked to cardiovascular diseases, such as myocardial infarction and heart failure (21, 23, 24). Their predictive value for CVD may be explained by the observation that, in the presence of inflammation, neutrophils engage in distinct defensive mechanisms such as degranulation, phagocytosis, reactive oxygen species production, and formation of neutrophil extracellular traps (NETs) (34). By enhancing systemic inflammation and engaging with endothelial cells and platelets, these mediators facilitate immune thrombosis, ultimately driving atherosclerosis and cardiovascular disease (CVD) (34, 35). Conversely, lymphocytes act as modulators of inflammation and confer resistance against atherosclerosis (36). Reduced lymphocyte counts have been linked to adverse outcomes in CVD patients, encompassing heart failure, chronic ischemic cardiopathy, and acute coronary syndromes (37, 38). In this context, our study further suggests that SII and SIRI may serve as valuable indicators for assessing LVH in patients with HUA (**Figure 1**).

In this study, we carefully controlled for potential confounders by excluding patients with pre-existing hypertension, diabetes, or advanced renal dysfunction (CKD stage >3) prior to admission (**Supplementary Figure 1**). Despite these exclusions, our findings consistently demonstrated that patients with HUA who developed LVH exhibited significantly higher SII and SIRI levels, suggesting that systemic inflammation may play an important role in myocardial remodeling (**Supplementary Table 1**). Multivariable logistic regression and subtype analyses, adjusting for demographic characteristics, lifestyle factors, and major clinical variables, both SII and SIRI levels remained independently and significantly associated with an increased risk of LVH in HUA patients (**Table 2**), consisting with previous studies (21, 23). Nonlinear modeling using restricted cubic spline and threshold effect analyses (**Figure 1**) revealed a clear dose–response relationship between SII and LVH risk. The inflection point was approximately 399.41 × 10^9^ cells/L (lnSII = 5.99), above which the probability of LVH rose sharply. Intriguingly, this threshold is substantially lower than the SII cut-off of about 1104.78 × 10^9^ cells/L reported by Zheng et al. as predictive of incident heart failure in a general population (24). From a clinical perspective, these findings support the concept that earlier identification of elevated SII and timely intervention in HUA patients could be important for preventing the development of LVH and potentially subsequent progression to overt heart failure. Nevertheless, cross-study comparisons should be interpreted cautiously, and the SII threshold identified in our analysis should be regarded as exploratory until validated in independent HUA cohorts. Furthermore, unsupervised clustering based on the SII–SIRI pattern (**Supplementary Figure 2**) identified a high-inflammation phenotype that was strongly associated with an elevated risk of LVH, emphasizing that ventricular remodeling is driven not by isolated biomarkers but by an integrated systemic inflammatory milieu. Exploratory mediation analysis further indicated that SII and SIRI partially mediated the relationship between serum uric acid and cardiac structural alterations, supporting a potential pathophysiological cascade of “HUA-inflammation-LVH”. Collectively, these findings strengthen the hypothesis that inflammation acts as a key intermediary linking hyperuricemia to adverse cardiac remodeling, offering potential targets for early prevention and intervention.

Although this study employed a relatively large sample size, strict inclusion and exclusion criteria, and comprehensive adjustment for multiple covariates to minimize potential confounding bias, several limitations should be acknowledged. Firstly, this was a single-center retrospective study, and participants were primarily recruited from hospitalized patients at our institution, which may have introduced selection and information biases. In particular, we applied strict exclusion criteria, removing individuals with pre-existing hypertension, diabetes, or CKD stage > 3. While this approach reduced confounding by comorbidities, it also limits the external promotion of the results. Moreover, LVH was defined using sex-specific LVMI thresholds, which may have contributed to the higher proportion of women in the LVH group despite their lower absolute LVMI and inflammatory indices compared with men. Thus, part of the observed sex difference in LVH prevalence may be attributable to the diagnostic criteria themselves rather than a purely biological excess risk. Furthermore, although our mediation analysis suggested that systemic inflammatory indices may partially mediate the association between serum urate and LVH, the retrospective cross-sectional design precludes us from establishing a clear temporal sequence among exposure, mediator, and outcome. Mediation analysis relies on several strong assumptions, including correct model specification, absence of unmeasured confounding of the exposure–outcome, exposure–mediator, and mediator–outcome relationships, and no exposure-induced mediator–outcome confounding. These assumptions are unlikely to be fully satisfied in an observational hospital-based study. Therefore, our mediation results should be interpreted as hypothesis-generating, providing a statistical decomposition of associations rather than definitive evidence of causal pathways. Finally, although SII/SIRI showed potential in predicting or evaluating LVH, its clinical applicability should be further validated in large-scale, multicenter, prospective cohort studies. Future studies could be improved in several aspects. Firstly, incorporating imaging parameters such as cardiac magnetic resonance T1 mapping or echocardiographic strain imaging may allow for more precise assessment of myocardial structural and functional remodeling. Secondly, population-based cohort studies are warranted to clarify the causal relationships between inflammatory markers and LVH in individuals with hyperuricemia, and to develop multi-marker predictive models incorporating metabolic parameters. Thirdly, mechanistic studies should be conducted to elucidate the signaling pathways and molecular mechanisms through which SII/SIRI mediate cardiac remodeling, thereby providing new theoretical insights for the early identification and intervention of HUA-related LVH.

## 5 Conclusion

In summary, this retrospective cross-sectional study provides evidence that SII and SIRI are positively associated with LVH in patients with HUA. In addition, the study connects HUA to LVH via inflammatory pathways, providing mechanistic insight. Nonetheless, given the limitations of this study, experimental validation in animal models and additional prospective research are warranted.

## Data Availability

The raw data supporting the conclusions of this article will be made available by the authors, without undue reservation.

## Supplementary material

Supplementary Figure 1 Flowchart of patient selection. LVH, left ventricular hypertrophy; CKD, chronic kidney disease.

Supplementary Figure 2 K-means clustering of patients based on lnSII and lnSIRI levels. Two distinct subgroups were identified, representing low- and high-inflammation patterns.

Supplementary Figure 3 Histogram. BMI body mass index, SBP, systolic blood pressure; DBP, diastolic blood pressure; SII, systemic immune-inflammation index; SIRI, systemic inflammation response index; ALT, alanine aminotransferase; AST, aspartate aminotransferase; FBG, fasting blood glucose; eGFR, estimated glomerular filtration.

Supplementary Figure 4 Q-Q plot. BMI, body mass index; SBP, systolic blood pressure; DBP, diastolic blood pressure; SII, systemic immune-inflammation index; SIRI, systemic inflammation response index; ALT, alanine aminotransferase; AST, aspartate aminotransferase; FBG, fasting blood glucose; eGFR, estimated glomerular filtration.
