# Supplementary figures and images for: Systemic inflammatory indices mediate the association between hyperuricemia and left ventricular hypertrophy: evidence from a single-center retrospective cross-sectional study
Source: Front Endocrinol (Lausanne). 2026 Jan 5;16:1742938. doi: 10.3389/fendo.2025.1742938 (PMC12813693; doi:10.3389/fendo.2025.1742938)

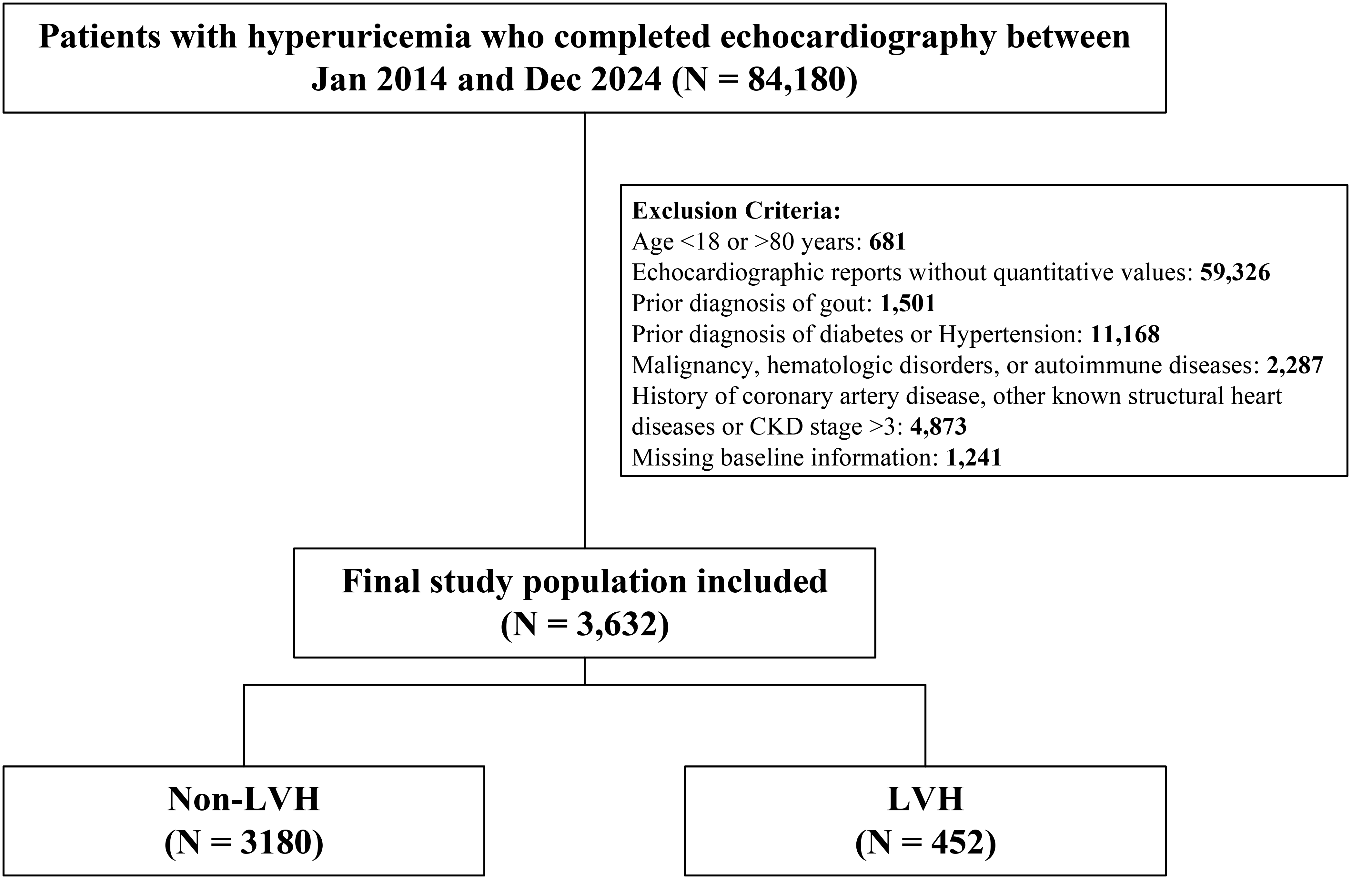

Supplement: Supplementary Figure 1 — Flowchart of patient selection. LVH, left ventricular hypertrophy; CKD, chronic kidney disease. [file Image1.tif]

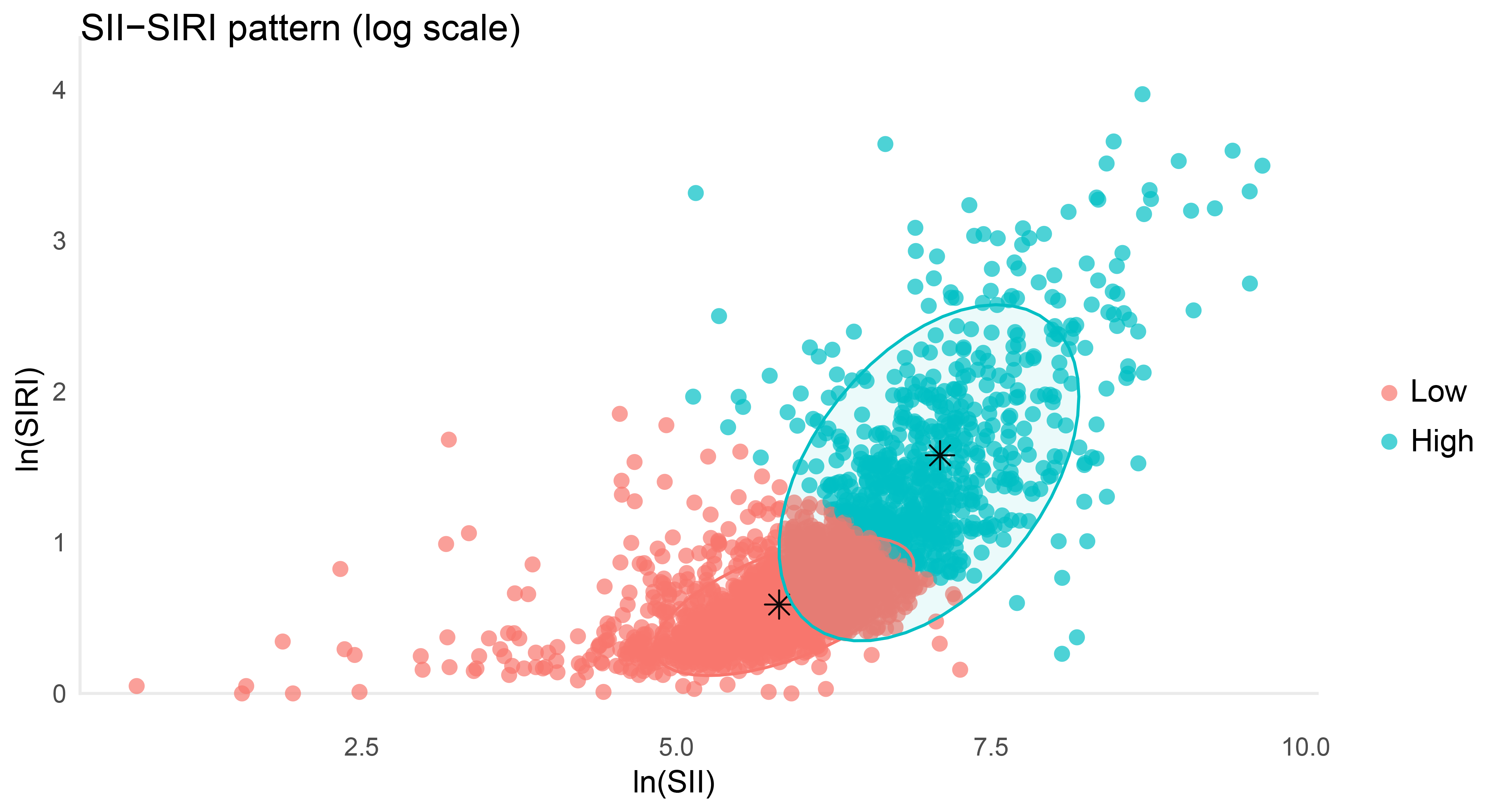

Supplement: Supplementary Figure 2 — K-means clustering of patients based on lnSII and lnSIRI levels. Two distinct subgroups were identified, representing low- and high-inflammation patterns. [file Image2.tif]
