# Supplementary material for: Systemic inflammatory indices mediate the association between hyperuricemia and left ventricular hypertrophy: evidence from a single-center retrospective cross-sectional study
Source: Front Endocrinol (Lausanne). 2026 Jan 5;16:1742938. doi: 10.3389/fendo.2025.1742938 (PMC12813693; doi:10.3389/fendo.2025.1742938)
Supplement: Supplementary file 5 [file Table1.docx]

| Supplement Table 1 Echocardiographic characteristics of participants based on subgroups of SII-SIRI pattern/SII/SIRI levels | | | | | | | | | | | |
| --- | --- | --- | --- | --- | --- | --- | --- | --- | --- | --- | --- |
| **Characteristics** | **SII-SIRI pattern** | | | **SII levels (10^9^ cells/L)** | | | | **SIRI levels (10^9^ cells/L)** | | | |
|  | **Low (n = 2955)** | **High (n = 677)** | **P** | **Low (n = 2179)** | **Medium (n = 726)** | **High (n = 727)** | **P** | **Low (n = 2179)** | **Medium (n = 726)** | **High (n = 727)** | **P** |
| AO, cm, Mean ± SD | 2.39 ± 0.42 | 2.37 ± 0.18 | **0.153** | 2.39 ± 0.47 | 2.39 ± 0.17 | 2.37 ± 0.18 | 0.460 | 2.39 ± 0.47 | 2.39 ± 0.17 | 2.37 ± 0.18 | 0.446 |
| LA, cm, Mean ± SD | 3.61 ± 0.44 | 3.63 ± 0.57 | 0.517 | 3.62 ± 0.45 | 3.60 ± 0.44 | 3.62 ± 0.55 | 0.383 | 3.60 ± 0.43 | 3.64 ± 0.46 | 3.65 ± 0.58 | **0.013** |
| LVEDD, cm, Mean ± SD | 4.60 ± 0.37 | 4.59 ± 0.47 | 0.496 | 4.61 ± 0.37 | 4.58 ± 0.38 | 4.58 ± 0.46 | 0.166 | 4.60 ± 0.36 | 4.60 ± 0.39 | 4.60 ± 0.47 | 0.964 |
| IVS, cm, Mean ± SD | 1.04 ± 0.14 | 1.06 ± 0.18 | 0.001 | 1.03 ± 0.14 | 1.05 ± 0.14 | 1.05 ± 0.17 | **<0.001** | 1.03 ± 0.13 | 1.05 ± 0.15 | 1.06 ± 0.17 | **<0.001** |
| LVPWT, cm, Mean ± SD | 0.96 ± 0.11 | 0.97 ± 0.13 | 0.018 | 0.95 ± 0.11 | 0.96 ± 0.11 | 0.96 ± 0.13 | 0.159 | 0.95 ± 0.11 | 0.96 ± 0.11 | 0.97 ± 0.13 | **<0.001** |
| EF, %, Mean ± SD | 0.63 ± 0.04 | 0.61 ± 0.06 | <0.001 | 0.63 ± 0.04 | 0.62 ± 0.04 | 0.61 ± 0.05 | **<0.001** | 0.63 ± 0.04 | 0.63 ± 0.04 | 0.61 ± 0.06 | **<0.001** |
| LVMI, g/m², Mean ± SD | 86.83 ± 20.01 | 90.66 ± 25.00 | <0.001 | 86.73 ± 20.06 | 87.98 ± 20.23 | 89.56 ± 24.49 | **0.006** | 86.02 ± 19.49 | 88.57 ± 21.77 | 91.10 ± 24.26 | **<0.001** |
