# Supplementary material for: Systemic inflammatory indices mediate the association between hyperuricemia and left ventricular hypertrophy: evidence from a single-center retrospective cross-sectional study
Source: Front Endocrinol (Lausanne). 2026 Jan 5;16:1742938. doi: 10.3389/fendo.2025.1742938 (PMC12813693; doi:10.3389/fendo.2025.1742938)
Supplement: Supplementary file 6 [file Table2.docx]

Supplement Table 2 Multicollinearity test for multivariable models (Based on Model 3)

|  | SII-SIRI pattern | Age | Gender | Smoking | Drinking | BMI | SBP | DBP | Crea | serum_urate | FBG | TG |
| --- | --- | --- | --- | --- | --- | --- | --- | --- | --- | --- | --- | --- |
| VIF | 1.048 | 1.149 | 1.375 | 1.637 | 1.573 | 1.197 | 1.659 | 1.641 | 1.081 | 1.062 | 1.075 | 1.141 |

|  | lnSII | Age | Gender | Smoking | Drinking | BMI | SBP | DBP | Crea | serum_urate | FBG | TG |
| --- | --- | --- | --- | --- | --- | --- | --- | --- | --- | --- | --- | --- |
| VIF | 1.018 | 1.148 | 1.372 | 1.637 | 1.575 | 1.196 | 1.657 | 1.634 | 1.075 | 1.045 | 1.075 | 1.141 |

|  | lnSIRI | Age | Gender | Smoking | Drinking | BMI | SBP | DBP | Crea | serum_urate | FBG | TG |
| --- | --- | --- | --- | --- | --- | --- | --- | --- | --- | --- | --- | --- |
| VIF | 1.061 | 1.146 | 1.371 | 1.639 | 1.575 | 1.195 | 1.658 | 1.641 | 1.079 | 1.076 | 1.074 | 1.141 |

|  | GVIF | Df | GVIF^(1/(2*Df)) |
| --- | --- | --- | --- |
| SII_Group | 1.039 | 2 | 1.01 |
| Gender | 1.376 | 1 | 1.173 |
| Smoking | 1.637 | 1 | 1.28 |
| Drinking | 1.573 | 1 | 1.254 |
| Age | 1.149 | 1 | 1.072 |
| BMI | 1.198 | 1 | 1.094 |
| SBP | 1.66 | 1 | 1.288 |
| DBP | 1.637 | 1 | 1.279 |
| Crea | 1.078 | 1 | 1.039 |
| serum_urate | 1.053 | 1 | 1.026 |
| FBG | 1.075 | 1 | 1.037 |
| TG | 1.141 | 1 | 1.068 |
|  | GVIF | Df | GVIF^(1/(2*Df)) |
| SIRI_Group | 1.061 | 2 | 1.015 |
| Gender | 1.373 | 1 | 1.172 |
| Smoking | 1.641 | 1 | 1.281 |
| Drinking | 1.573 | 1 | 1.254 |
| Age | 1.146 | 1 | 1.071 |
| BMI | 1.197 | 1 | 1.094 |
| SBP | 1.66 | 1 | 1.289 |
| DBP | 1.643 | 1 | 1.282 |
| Crea | 1.082 | 1 | 1.04 |
| serum_urate | 1.071 | 1 | 1.035 |
| FBG | 1.074 | 1 | 1.036 |
| TG | 1.142 | 1 | 1.068 |

**Abbreviations**: VIFs = variance inflation factors, GVIF = generalized variance inflation factor, Df = degrees of freedom, BMI = body mass index, SBP = systolic blood pressure, DBP = diastolic blood pressure, SII = systemic immune-inflammation index, SIRI = systemic inflammation response index, FBG = fasting blood glucose, TG = triglycerides.
